# *In Vivo* Labeling and Detection of Circulating Tumor Cells in Mice Using OTL38

**DOI:** 10.1007/s11307-024-01914-0

**Published:** 2024-04-09

**Authors:** Joshua Pace, Jane J. Lee, Madduri Srinivasarao, Shivakrishna Kallepu, Philip S. Low, Mark Niedre

**Affiliations:** 1 Department of Bioengineering, Northeastern University, Boston, MA 02115 USA; 2 Department of Chemistry, Purdue University, West Lafayette, IN 047906 USA

**Keywords:** Fluorescence, *In vivo* flow cytometry, Diffuse imaging, Folate receptor, Circulating tumor cells (CTCs), Fluorescence guided surgery (FGS)

## Abstract

**Purpose:**

We recently developed an optical instrument to non-invasively detect fluorescently labeled circulating tumor cells (CTCs) in mice called ‘Diffuse *in vivo* Flow Cytometry’ (DiFC). OTL38 is a folate receptor (FR) targeted near-infrared (NIR) contrast agent that is FDA approved for use in fluorescence guided surgery of ovarian and lung cancer. In this work, we investigated the use OTL38 for *in vivo* labeling and detection of FR + CTCs with DiFC.

**Procedures:**

We tested OTL38 labeling of FR + cancer cell lines (IGROV-1 and L1210A) as well as FR- MM.1S cells in suspensions of Human Peripheral Blood Mononuclear cells (PBMCs) *in vitro*. We also tested OTL38 labeling and NIR-DIFC detection of FR + L1210A cells in blood circulation in nude mice *in vivo.*

**Results:**

62% of IGROV-1 and 83% of L1210A were labeled above non-specific background levels in suspensions of PBMCs *in vitro* compared to only 2% of FR- MM.1S cells. L1210A cells could be labeled with OTL38 directly in circulation *in vivo* and externally detected using NIR-DiFC in mice with low false positive detection rates.

**Conclusions:**

This work shows the feasibility of labeling CTCs *in vivo* with OTL38 and detection with DiFC. Although further refinement of the DiFC instrument and signal processing algorithms and testing with other animal models is needed, this work may eventually pave the way for human use of DiFC.

## Introduction

Cancer metastasis is a leading cause of death in humans [1]. One of the main pathways of metastasis is via the blood circulatory system (“hematogenous metastasis”) wherein tumor cells intravasate into the vasculature from the primary tumor and travel to distant organs and tissues [2–4]. Circulating tumor cells (CTCs) and multicellular circulating tumor cell clusters (CTCCs) are therefore of great interest to clinicians and cancer researchers [5–8].

The gold standard for quantification and characterization of CTCs is liquid biopsy, wherein small blood samples are drawn from a small animal or human patient. CTCs are preferentially isolated by depletion of red blood cells and captured using cell phenotype differences such as cell surface receptor expression, size, or mechanical properties [9, 10].

Recent evidence shows that this methodology may lead to poor quantification of CTC numbers for several reasons. First, the blood volume drawn is small compared to the overall peripheral blood (PB) volume (e.g. 7.5 mL used for the CellSearch Clinical CTC assay compared to 5 L human PB volume) [11]. Even with stable CTC numbers, the small fractional blood volume sampled yields poor quantitative accuracy and rare CTCs may be missed entirely [12, 13]. Second, the number of CTCs in circulation is frequently far from stable; CTC counts can vary over relatively short timescales and with circadian patterns [14, 15]. Moreover, CTC numbers in drawn blood samples have been shown to vary with the location of the blood draw from the body relative to the tumor and major draining organs [16]. Therefore, several researchers have studied alternative non-invasive optical methods for enumerating CTCs [17–20].

Our group developed Diffuse *in vivo* Flow Cytometry (DiFC) as a novel method for fluorescence detection and enumeration of CTCs in small animal models of cancer metastasis [21]. Compared to microscopy-based methods for enumerating CTCs, DiFC uses an external optical device to probe relatively deep tissues and large blood vessels with highly scattered light. Careful optical design and algorithmic processing allows detection and counting of fluorescently-labeled CTCs. We have previously used DiFC to study CTC dissemination serially, over time in mouse xenograft models with fluorescent protein expressing cancer cells [12, 22, 23].

With respect to potential human translation of DiFC, we also showed that it would in principle be possible to detect and count fluorescently-labeled CTCs to a depth of 2–4 mm using near infrared (NIR) fluorophores in biological tissue [24, 25]. In human anatomy, there are several potential large blood vessels such as the radial artery in the wrist that could be used to sample large amounts of circulating PB (> 100 mL per minute) [26]. To this end, we recently built a DiFC prototype compatible with NIR fluorophores [27].

A major challenge in human translation of DiFC is fluorescence labeling of CTCs since, unlike in mice, CTCs would need to be labeled directly *in vivo*. Our work and others have demonstrated that it is feasible to label specific populations of circulating cells with receptor-targeted contrast agents, although to-date these have been limited to pre-clinical studies in mice [18, 28–30]. Our group showed that a FITC-based probe EC-17 could be used to label CTCs *in vivo*. However, due to the large optical attenuation of blue-green light in biological tissue, and the lack of FDA approval, there was minimal direct future potential for human translation [30].

However, in recent years there have been major technological and regulatory advances in the area of molecularly-targeted, cancer-specific NIR fluorophores in for human use [31]. These contrast agents are overwhelmingly developed for the field of fluorescence guided surgery (FGS) in humans, and we hypothesize that the specificity for cancer tumors may translate to labeling CTCs. Notably, OTL38 (Cytalux) is a folate-receptor alpha (FR $\alpha$) targeted NIR molecular contrast agent that was recently FDA-approved in the United States for lung and ovarian cancer [32]. FR $\alpha$ is over-expressed in many cancer types including breast, lung, ovarian, and prostate, and has low expression in normal tissues and immune cells in the blood [30, 33–37]. OTL38 is also optically compatible with the absorption and emission spectra of our NIR DiFC system [27].

In this work, we used OTL38 to label FR $\alpha$-expressing tumor cells in complex suspensions of peripheral blood mononuclear cells (PBMCs). We also labeled FR expressing tumor cells while in circulation in the bloodstream *in vivo* for the first time. We showed that these tumor cells could be detected non-invasively using our NIR DiFC system with very low rates of false positive detections. In combination with our earlier work showing the feasibility of performing DiFC in human-scale geometries, this work paves the way for potential clinical translation of the DiFC for detection of CTCs.

## Materials and Methods

### NIR-DiFC Instrument

**Fig. 1:**
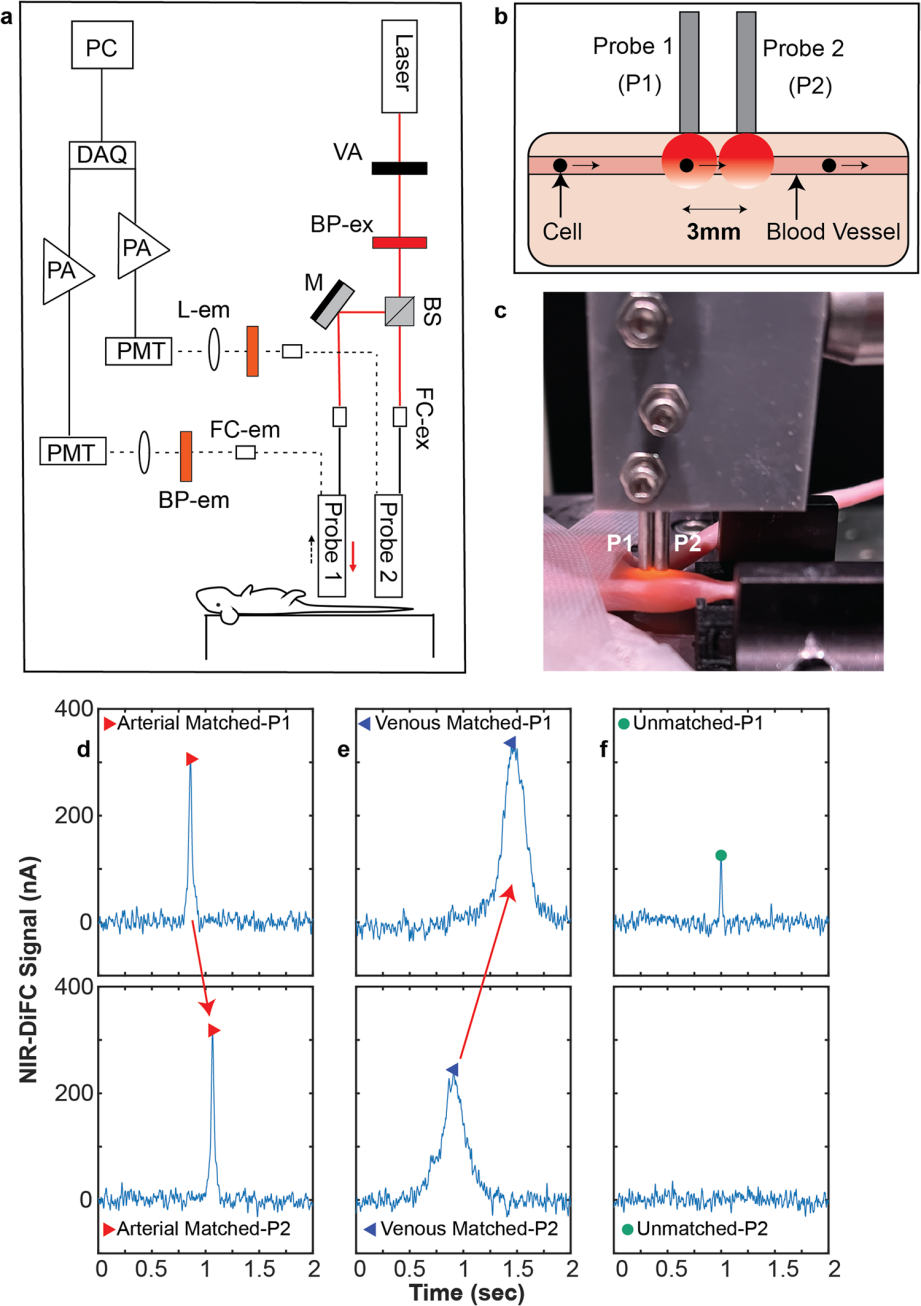
The NIR-DiFC instrument and working principle **a** Instrument schematic, reproduced with permission from Pace et al*.* [27]. **b** As fluorescently-labeled cells travel in a blood vessel under the DiFC system, fluorescence signal is collected by the optical fiber probe assemblies P1 and P2. **c** Photograph of the NIR-DiFC optical fiber probes placed on the skin over the great saphenous vessels in the hindlimb of a nude mouse. The 3 mm separation between P1 and P2 allows for cell “matching” of cells traveling in the blood vessel. **d** Cells traveling in the artery cause a detection in P1 before P2, whereas cells travelling in the vein **e** cause a detection in P2 before P1. **f** Unmatched cells (due to cells moving in other blood vessels, noise, or motion artifacts) are discarded by the algorithm.

The schematic of NIR-DiFC is shown in Fig. 1a [27]. The light source is a tunable pulsed laser (Mai Tai XF-1, Spectra Physics, Santa Clara, CA) with excitation wavelength set to 770 nm. The power is adjusted with a variable attenuator (VA) before it is passed through a 766/13 nm bandpass clean up filter (BP-ex; FF01-766/13-25, IDEX Health and Science LLC, Rochester, NY). The light is then split into two beams with a beam splitter (BS; 49005, Edmund Optics, Barrington, NJ) before being coupled with a collimation package (FC-ex; F240SMA-780, Thorlabs Inc., Newton, NJ) into source fibers of the optical fiber probe assemblies. For a detailed explanation of the optical fiber probes, refer to [27]. The light power at the sample is set to 25 mW. The output of the probe collection fibers is collimated (FC-em; F240SMA-780, Thorlabs) and the light is passed through an 810/10 nm bandpass emission filter (BP-em; FF01-810/10-25, IDEX Health and Science LLC) before being focused on to the surface of a photomultiplier tube (PMT; H10721-20, Hamamatsu, Bridgewater, NJ) with a 30 mm focal length lens (L-em; 67543, Edmund Optics). The PMTs are powered by a power supply (C10709; Hamamatsu). Output signals from the PMTs are filtered with an electronic 100 Hz low pass filter, amplified with a low-noise current pre-amplifier (PA; SR570, Stanford Research Systems, Sunnyvale, CA), and then acquired with a data acquisition board (USB-6343 BNC; National Instruments, Austin, TX).

### NIR-DiFC Scanning and Data Analysis

DiFC scanning and data analysis was performed as we described in our previous work [27]. Briefly, the optical fiber probe assemblies probe 1 (P1) and probe 2 (P2) are placed on the surface of the skin as shown in Fig. 1b. In the work here, P1 and P2 were aligned on the hindleg of the mouse, approximately above the great saphenous blood vessels (shown in Fig. 1c) or the ventral caudal vessels in the tail of the mouse. As fluorescently-labeled cells travel in the bloodstream they pass underneath P1 and P2. Excitation laser light results in a transient emitted fluorescence signal which is collected by fiber probe assembly. DiFC data from each probe was analyzed with custom written code in MATLAB using the approach described in detail previously:

1. The signal background (due to tissue autofluorescence and instrument background) is estimated as the local moving median value in a 5 s window. This is subtracted from the signal from each DiFC probe.
2. The resulting signal noise (standard deviation) is then calculated in a 1-min moving window.
3. Peak candidates were then identified as local maxima exceeding a threshold of four times the local noise, which gives a minimum SNR of 20*log_10_(4) = 12.04 dB.

The resulting peaks are then analyzed using a matching algorithm as follows. When the fiber probes are aligned above an artery a peak appears in the P1 (Fig. 1d) and then P2 with a delay corresponding to the transit time between the cells. Likewise, cells moving in the venous direction result in a detection in P2 followed by P1 (Fig. 1e). Cells moving in the venous direction frequently result in temporally wider peaks than the arterial direction due to their slower speed traveling through the DiFC probe field of view. In the case of cells traveling in a capillary bed, peaks are typically observed on one (but not both) probes as in Fig. 1f. Occasionally electronic noise or motion artifacts may also result in spurious unmatched peaks. These unmatched peaks are discarded from analysis, which results in a low operating false-alarm rate.

### Cancer Cell Lines

L1210A is an immortalized murine leukemia suspension cell line previously modified to over-express FR (Purdue University, West Lafayette, IN) [38–40]. IGROV-1 is an immortalized human ovarian cancer adherent cell line that naturally expresses FR (SCC203; Sigma-Aldrich, St. Louis, MO). MM.1S is an immortalized human multiple myeloma suspension cell line that does not express FR and was used as a control (CRL-2974, ATCC, Manassas, VA). All cell lines were cultured in RPMI 1640 folic acid deficient media (Gibco 27016021; ThermoFisher Scientific, Waltham, MA) supplemented with 10% fetal bovine serum (Gibco 16000044; ThermoFisher Scientific) and 1% penicillin/streptomycin (Gibco 15140122; ThermoFisher Scientific) and incubated at 37 °C with 5% CO_2_. For all experiments, cells were first collected from T-75 tissue culture treated flasks (FB012937; Thermofisher Scientific). For the adherent cell line IGROV-1, the cell culture media was aspirated and then 6 mL TrypLE Express (Gibco 12604021; ThermoFisher Scientific) added for 5 min to get the cells into suspension.

### OTL38 Folate Receptor Targeted Molecular Probe

OTL38 (On Target Laboratories, West Lafayette, IN) is a small molecule NIR fluorescent dye (MW 1326.49 g/mol) that targets FR⍺ cell surface receptors. OTL38 is FDA approved for the use in fluorescence guided surgery of ovarian and lung cancer under the name Cytalux [32, 41, 42]. OTL38 uses S0456 dye [similar fluorescence spectrum to indocyanine green (ICG)] is conjugated to a folate analog to and has 776 nm and 793 nm maximum excitation and emission wavelengths, respectively [43].

### Labeling of Cells with OTL38 *In Vitro*

Experiments where cancer cells were labeled in culture *in vitro* are subsequently referred to in this manuscript as “prelabeled cells” (as opposed to cells labeled in while in blood circulation *in vivo*). Approximately 10^6^ cells were suspended in phosphate-buffered saline (PBS) (Gibco 10010049; ThermoFisher Scientific) with 2% FBS (30-2020; ATCC) added for each experiment. 200 nM (20 μL of 10 μM stock) of OTL38 was added to the suspensions at 37 °C with 5% CO_2_ for 1 h. Cells were then washed twice with PBS before further experiments.

### Labelling of Cells with CellTrace CFSE Fluorophore

In some experiments, 10^6^ cancer cell suspensions in PBS were also stained with a green fluorophore, CellTrace CFSE (Invitrogen C34554; ThermoFisher Scientific) according to the manufacturer’s instructions prior to OTL38 labeling as above. As we show, this allowed us to differentiate cancer cells from other cell types in complex suspensions of Peripheral Blood Mononuclear Cells [PBMCs, (PCS-800-011; ATCC)] or peripheral blood.

### Labeling of FR + Cells with OTL38 in Suspensions of PBMCs *In Vitro*

To study specificity of OTL38 for cancer cells in the presence of other types of blood cells that may bind or scavenge OTL38 we studied complex suspensions of human PBMCs (which include dendritic cells, monocytes, and lymphocytes). 10^6^ PBMCs and 10^4^ CellTrace CFSE stained cancer cells were suspended in 1 mL of PBS with 2% FBS added. OTL38 was added to the suspension as described in Sect. "Labeling of Cells with OTL38 In Vitro". To introduce competitive binding (blocking) of OTL38, some suspensions of PBMCs and IGROV-1 cells were first co-incubated with 10 µM free folic acid (F7876; Sigma-Aldrich) for 30 min before addition of OTL38. All cell solutions were washed with fresh PBS twice and then analyzed by fluorescence Flow Cytometry (see Sect. "Flow Cytometry"). All samples were repeated at least *N* = 6 times (least 2 repeats and 3 replicates) with a minimum of 100,000 fluorescent count events.

### NIR-DiFC Detection of Prelabeled L1210A Cells in Mice

All mice were handled in accordance with Northeastern University’s Institutional Animal Care and Use Committee (IACUC) policies on animal care. Animal experiments were carried out under Northeastern University IACUC protocol #21-0412R. All experiments and methods were performed with approval from and in accordance with relevant guidelines and regulations of Northeastern University IACUC.

First, to determine detectability of CTCs well-labeled with OTL38*,* we intravenously *i.v.* injected prelabeled L1210A cells in the tail vein of nude mice. L1210A cells were first double labeled with OTL38 and Cell Trace CFSE *in vitro* and were suspended in 100 μL of cell culture media. Cell suspensions were injected *i.v.* via the tail vein of 6–8-week-old female Athymic nude mice (Nu/Nu/553; Jackson Laboratory, Bar Harbor, ME). NIR-DiFC was preformed 10 min after injection on the saphenous vessels for 60 min for each mouse (*N* = 3). Following NIR-DiFC scanning, blood draws were performed by terminal cardiac puncture. For the blood collection, 200 µL Heparin (H3393-10KU; Sigma-Aldrich) was drawn into a 3 mL 27G syringe (309570; Becton, Dickinson and Company, Franklin Lakes, NJ) to prevent blood clotting. After removing as much peripheral blood as possible (> 500 µL), the peripheral blood and Heparin mixture was added to 0.5 mL K_3_ EDTA coated tubes (450475; Greiner Bio-One, Kremsmünster, Austria) for processing and cells were then analyzed by benchtop fluorescence Flow Cytometry (Sect. "Flow Cytometry").

### NIR-DiFC Detection of L1210A Cells Labeled with OTL38 in Circulation *In Vivo*

Next, to determine the feasibility of labeling CTCs while in circulation in the bloodstream *in vivo* (as opposed to in cell culture prior to injection), we sequentially injected L1210A cells and OTL38 in nude mice intravenously. We subsequently refer to these as “*in vivo* labeled cells” in this manuscript. L1210A cells were first labeled with CellTrace CFSE *in vitro* as above, suspended in 100μL of cell culture media and *i.v.* injected in of 6–8-week-old female Athymic nude mice (*N* = 3). After 5 min, 2.5 µg of OTL38 dissolved in 100 µL PBS was *i.v.* injected via the tail vein. After approximately 1 h, NIR-DiFC was performed on the hindlimb vessels for 60 min. As discussed in the results section below, this increased time before DiFC scanning was due to an increase in fluorescence background signal immediately after injecting free OTL38. After 60 min we drew blood samples and analyzed them using Flow Cytometry.

### Systemic OTL38 Clearance and NIR-DiFC Background

In a separate set of experiments, 6–8-week-old female Athymic nude mice were injected via the tail vein with 2.5 µg OTL38 dissolved in 100 µL PBS. NIR-DiFC scanning was performed for 10 min at 3-,6-,9-,12-, 24-h post injection (*N* = 3). The background signal was recorded and normalized to a baseline value for each individual mouse.

### Flow Cytometry

Cell samples were analyzed using a benchtop Attune NXT flow cytometer (FC) (ThermoFisher Scientific). NIR fluorescence was collected using a 637 nm laser and a 780/60 nm emission filter. CellTrace CFSE green fluorescence was collected using a 488 nm laser and 530/30 nm emission filter. Samples were analyzed using FlowJo software and samples were gated for size and singlets based on corresponding cell populations. In cases where mouse blood samples were analyzed, red blood cells (RBCs) were first depleted by adding the samples to 2 mL 10 × RBC Lysis buffer (420,301; BioLegend, San Diego, CA) diluted to 1 × in 18 mL sterile water for 15 min. Suspensions were then washed twice with PBS and resuspended to a final volume of 3 mL PBS.

## Results

### Labeling of FR + Cells with Human PBMCs

**Fig. 2:**
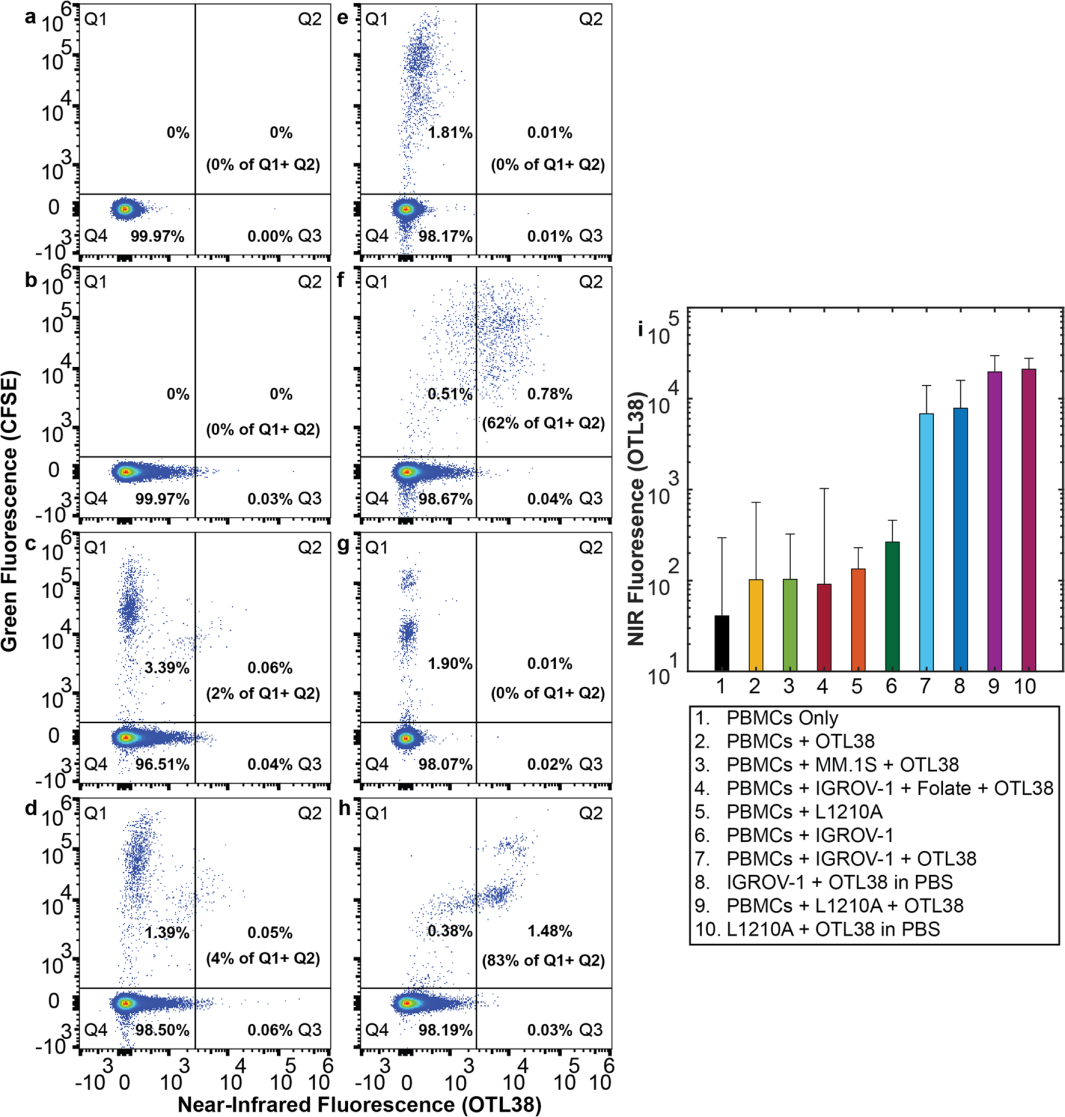
Flow Cytometry analysis of OTL38 labeling of FR + cancer cells in complex suspensions of human PBMCs. The green (CFSE) and NIR (OTL38) fluorescence for suspensions of **a** PBMCs only, **b** PBMCs incubated with OTL38, **c** FR- MM.1S cells suspended in PBMCs and labeled with OTL38, **d** FR + IGROV-1 cells suspended in PBMCs with 1000 × free folic acid and labeled with OTL38, **e** FR + IGROV-1 cells suspended in PBMCs only, **f** FR + IGROV-1 cells suspended in PBMCs and labeled with OTL38, **g** FR + L1210A cells suspended in PBMCs only, and **h** FR + L1210A cells suspended in PBMCs labeled with OTL38. **i** The mean OTL38 fluorescence intensity for the conditions (a-h) over *N* = 3 repetitions, showing high affinity of OTL38 for FR + cancer cells.

We first performed OTL38 labeling of cancer cells in suspensions of human PBMCs. Since human PBMCs are comprised of dendritic cells, monocytes, T cells, B cells, and NK cells, this provides a more realistic model of nonspecific uptake that may occur in whole blood *in vivo* [44]. Summarized in Fig. 2 are the results of FC analysis on these suspensions. In each panel Figs. 2a-h the vertical axes indicate green (CFSE) fluorescence, and the horizontal axes indicate NIR (OTL38) fluorescence. The NIR background level (vertical black line in each panel) was determined to be the threshold of detection of NIR-DiFC. This estimated threshold was based on previous work comparing the brightness of fluorescent microspheres ran on both NIR-DiFC and FC [27]. Four negative controls were performed as follows: the fluorescence of PBMC cells only are shown in Fig. 2a. Addition of OTL38 to PBMC suspensions yielded only a small amount of non-specific uptake as shown in Fig. 2b. These false positives are indicated in quadrant 3 (Q3) and represented 0.03 ± 0.01% of all events. This was likely due to macrophage scavenging of OTL38 as observed previous work [30, 45]. FR- MM.1S cells suspended in PBMCs and incubated with OTL38 (Fig. 2c) showed that only a small percentage (2 ± 0.5%) of cells labeled above background, shown in quadrant Q2. To test competitive binding of OTL38, free folic acid was added to samples of FR + IGROV-1 cells suspended in PBMCs prior to labelling with OTL38. As shown in Fig. 2d, free folic acid inhibited binding of OTL38 and only 4 ± 2% of cells were detected in Q2.

In contrast, FR + IGROV-1 cells suspended in PBMCs (Fig. 2e) and labeled with OTL38 (Fig. 2f) showed significant OTL38 binding, with 62 ± 8% of cells in Q2 above NIR background levels. Likewise, FR + L1210A cells suspended in PBMCs (Fig. 2g) and labeled with OTL38 (Fig. 2h), 83 ± 14% of cells above the background in Q2.

The NIR fluorescence data is summarized in Fig. 2i**,** where the mean OTL38 fluorescence intensity in each case is shown. Overall, we observed significant uptake and specificity for FR + cells, with an increase by a factor of 108 on average above controls. These are also consistent with previous studies with small molecule FR-targeted contrast agents showing high affinity for FR + cancer cells by our group and others [30, 42, 43].

### *In Vivo* Detection of OTL38 Prelabeled L1210A with NIR-DiFC and Flow Cytometry

**Fig. 3:**
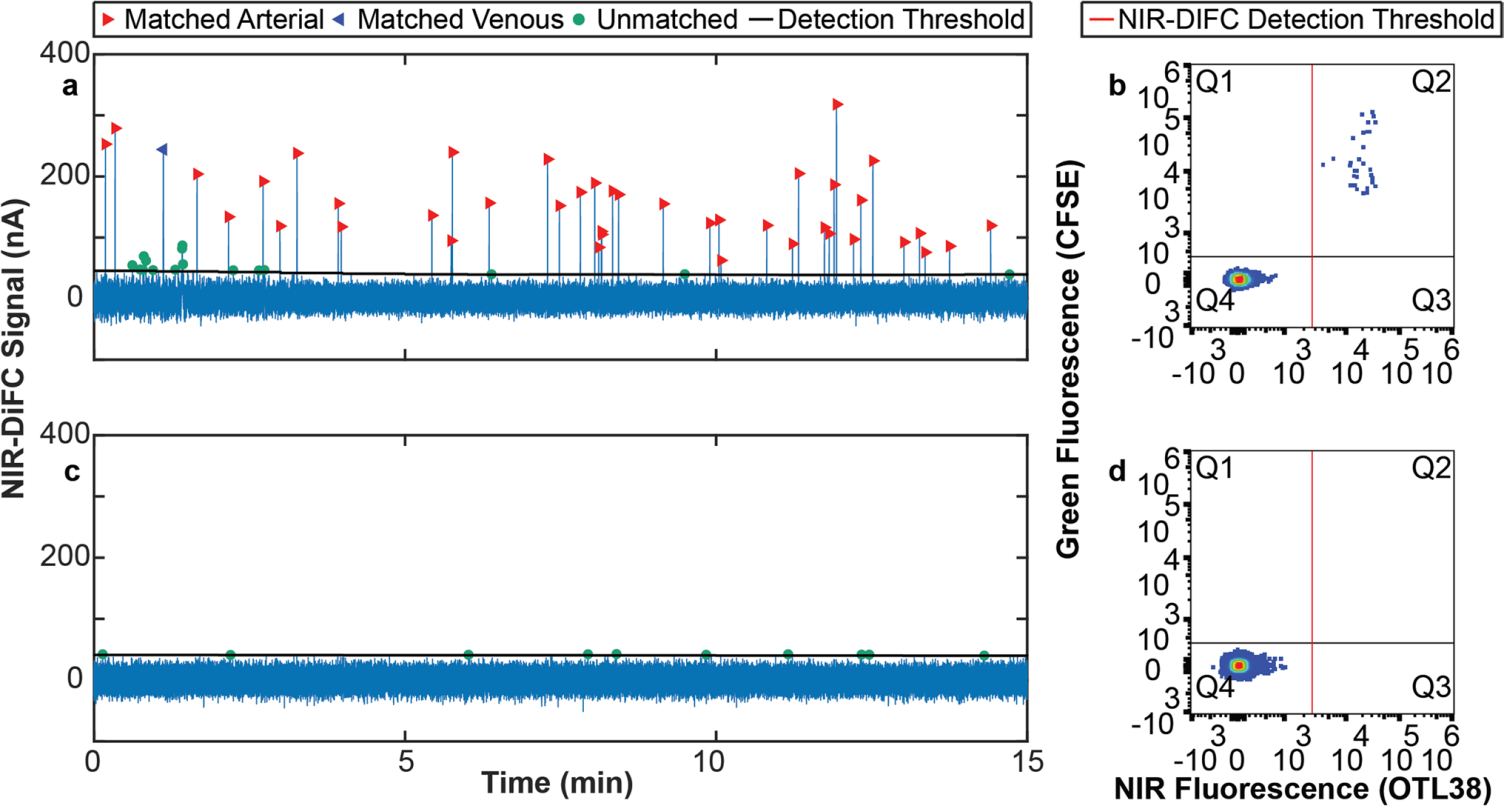
NIR-DiFC detection of OTL38 prelabeled L1210A cells *in vivo*. **a** Representative NIR-DiFC data from the hindleg of nude mice *in vivo*. Mice were injected *i.v.* with L1210A cells labeled with CFSE and OTL38 prior to injection (*N* = 3). Red arrowheads represent forward (arterial) matched cells, blue arrowheads represent reverse (venous) matched cells, and each green circles represent unmatched cell detections. **b** Fluorescence Flow Cytometry analysis of blood samples taken from mice after NIR-DiFC scanning confirmed the presence of CFSE (green) and OTL38 (NIR) labeled cells (Q2). **c** Representative NIR-DiFC data from an un-injected control mouse showing no forward or reverse detections **d** Fluorescence Flow Cytometry of blood samples from an un-injected control mouse confirmed no false positives (*N* = 3).

To test the brightness and NIR-DiFC detectability of L1210A cells labeled with OTL38, we first double-labeled cells with CFSE and OTL38 in culture *in vitro* and then injected 10^6^ cells *i.v.* via the tail vein of nude mice (*N* = 3). Most injected cells are cleared rapidly from circulation through the “first pass effect” through the lungs and liver so that only a small number L1210A cells remain in circulation after a few minutes [46–48]. After L1210A cell injection, we performed NIR-DiFC on the hindleg for approximately 1 h. Example background-subtracted data is shown in Fig. 3a. Each peak represents detection of a cell by NIR-DiFC. Further, each red arrows indicates a cell traveling in the forward direction with arterial flow between DiFC probes P1 and P2, whereas blue arrowheads represent cells traveling in the reverse, with venous flow between P2 and P1. Green circles represent unmatched peaks, which (as described in Sect. "NIR-DiFC Scanning and Data Analysis" above) may be attributed to motion or noise artifacts, or cells travelling in smaller blood vessels. Therefore, unmatched peaks were discarded from analysis.

Immediately after scanning, blood was collected from the mice via cardiac puncture. Blood samples were analyzed by benchtop Flow Cytometry (FC) as shown in Fig. 3b. Here, the green fluorescence (y-axis) and NIR fluorescence (x-axis) of the cell samples are shown. The cluster in the bottom left Q4 quadrant represents cells found in the blood. The double-positive (CFSE and OTL38) sub-population (Q2 quadrant) shows the injected L1210A cells that remained in circulation at the time of the blood draw. The estimated NIR-DiFC fluorescence detection threshold with our current design is indicated by the red vertical line.

From these data we determined that there were 60 ± 43 double (CFSE and OTL38)-labeled L1210A cells per mL blood remaining in circulation (*N* = 3 mice). Given that the average NIR-DiFC count rate (Fig. 3a) in the same mice was 3.6 ± 1.3 cells per minute, we estimate that the NIR-DiFC blood sampling rate in the saphenous artery was approximately 3.6 cells per minute / 60 cells per mL = 60 µL per minute. This is similar to the sampling rate of 48 µL per minute that we previously reported for the femoral artery in nude mice [30]. A blood flow rate for either the saphenous or femoral was not found in the literature.

Figure 3c shows 15 min of representative NIR-DiFC data collected from an un-injected control mouse. As shown, no false-positive matched cells were detected (as above, green symbols are un-matched peaks which are discarded as noise). After implementing the peak-matching signal processing algorithm, the false alarm rate for 3 h of scanning (with 3 separate mice, one hour each) was 0.03 cells/min, illustrating the stability of the NIR-DiFC signal. This is consistent with our previous DiFC studies in mouse models with the same cell line [30]. Likewise, Flow Cytometry analysis of blood samples collected post-scanning (Fig. 3d) showed that no double-labeled cells were present in quadrant Q2.

### *In Vivo* OTL38 Labeling and NIR DiFC Detection of L1210A Cells

**Fig. 4:**
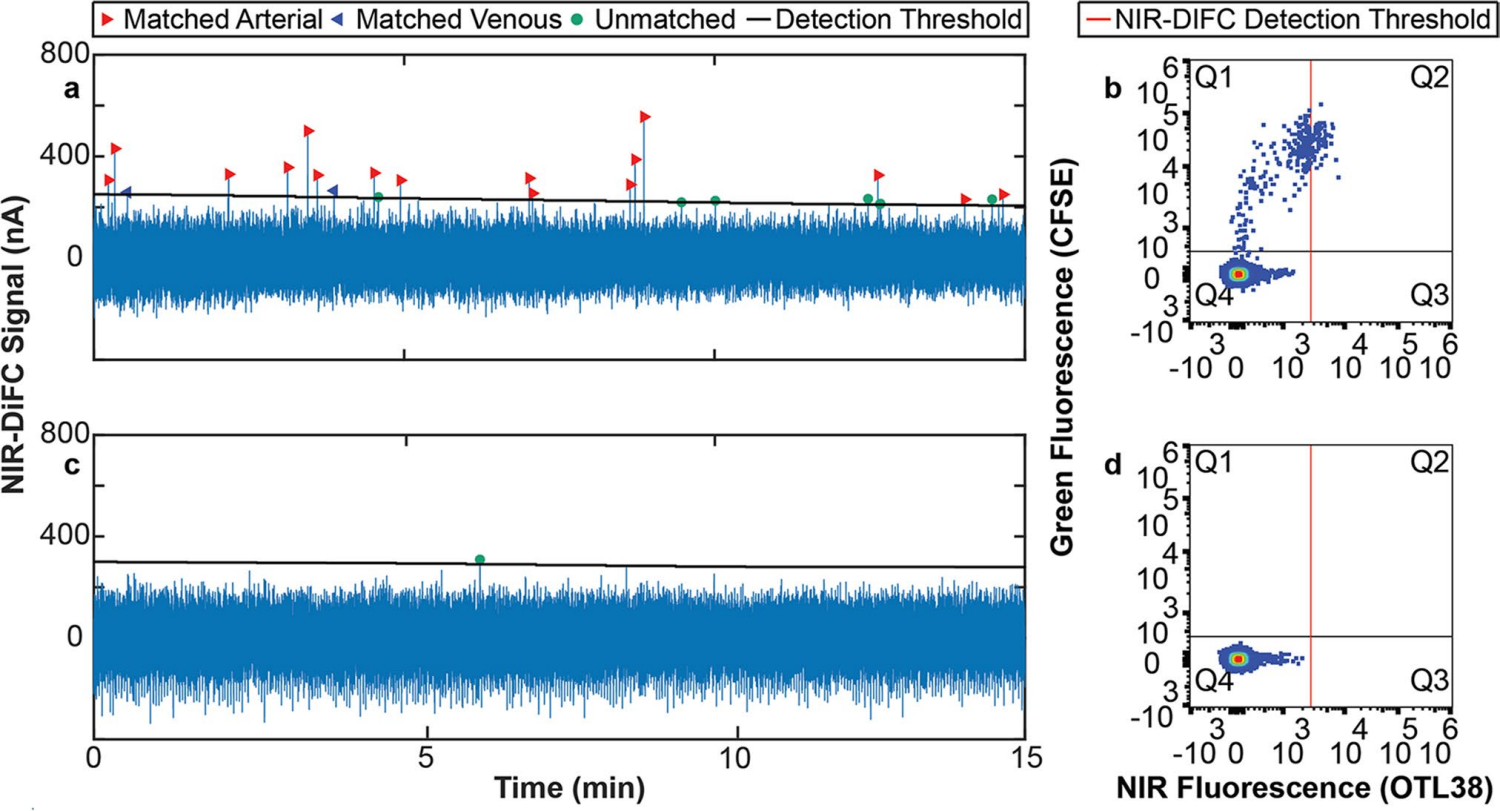
NIR-DiFC detection of L1210A cells labeled with OTL38 in circulation *in vivo.* **a** Representative NIR-DiFC data acquired approximately 1 h after CFSE-labeled L1210A and 2.5 µg OTL38 were separately injected via the tail vein *i.v.* (*N* = 3). **b** Fluorescence Flow Cytometry analysis of the blood samples after scanning indicating the presence of a labeled (Q2) sub-population of L1210A cells. **c** Representative NIR-DiFC data from control mice approximately 1 h after injection of 2.5 µg OTL38 only, showing no false positive matched cell detections. **d** Flow Cytometry of blood samples showing negligible labeling of blood cells with OTL38 (Q3).

We next labelled CTCs directly in circulation in the bloodstream with OTL38 (as opposed to labeling cells prior to injection). We first injected CFSE (green) labeled L1210A cells via the tail vein. After approximately 5 min, we injected 2.5 µg OTL38. Representative NIR DiFC data acquired from the hindleg beginning approximately 1 h after OTL38 injection is shown in Fig. 4a and multiple cell detections in the forward and reverse directions were recorded. As we discuss in more detail in Sect. "Comparison Between Prelabeled and In Vivo Labeled NIR-DiFC Detection Rate", we observed a larger background signal and corresponding background noise (after background subtraction) than with prelabeled cells, presumably due to the presence of unbound OTL38 in the blood or surrounding tissue. Following DiFC, blood samples were drawn and analyzed by fluorescence Flow Cytometry (Fig. 4b). Since L1210A cells were prelabeled with CFSE prior to injection, cells in quadrant Q2 indicate OTL38-labeled L1210A cells (true positive), whereas cells in Q1 indicate L1210A cells with low OTL38 labeling (false negative). Although OTL38 uptake by cells in circulation was (unsurprisingly) lower than cells labeled in culture (Fig. 3b), these data confirmed that it is indeed feasible to label an FR + cell with a NIR molecular contrast agent directly *in vivo* with sufficient brightness for external optical detection with DiFC.

Equally important, injection of 2.5 µg of OTL38 without L1210A cells resulted in minimal false positive signals. 15 min of representative data is shown in Fig. 4c**.** The overall false alarm rate was again 0.03 cells/min for 3 h of scanning (with 3 separate mice, one hour each). Fluorescence Flow Cytometry of blood samples (Fig. 4d) showed very little uptake of OTL38 by other blood. In total we observed only 3 cells in quadrant Q3 (false positive) in 3 mice. In combination, Fig. 4c, d indicate very high specificity of OTL38 for FR + circulating cells, with very low corresponding NIR-DiFC false alarm rate.

We also note that we delayed the start of NIR-DiFC scanning for approximately 1 h after injection of OTL38 due to the observed increased background fluorescence signal after injection compared to the baseline. Our previous work indicted that the number of L1210A cells in circulation was statistically unchanged in the 2-h period following injection, so this longer interval likely did not significantly contribute to the lower DiFC count rate observed for the *in vivo* labeling case [27, 30]. The implications of this are discussed in the next section.

### Comparison Between Prelabeled and *In Vivo* Labeled NIR-DiFC Detection Rate

**Fig. 5:**
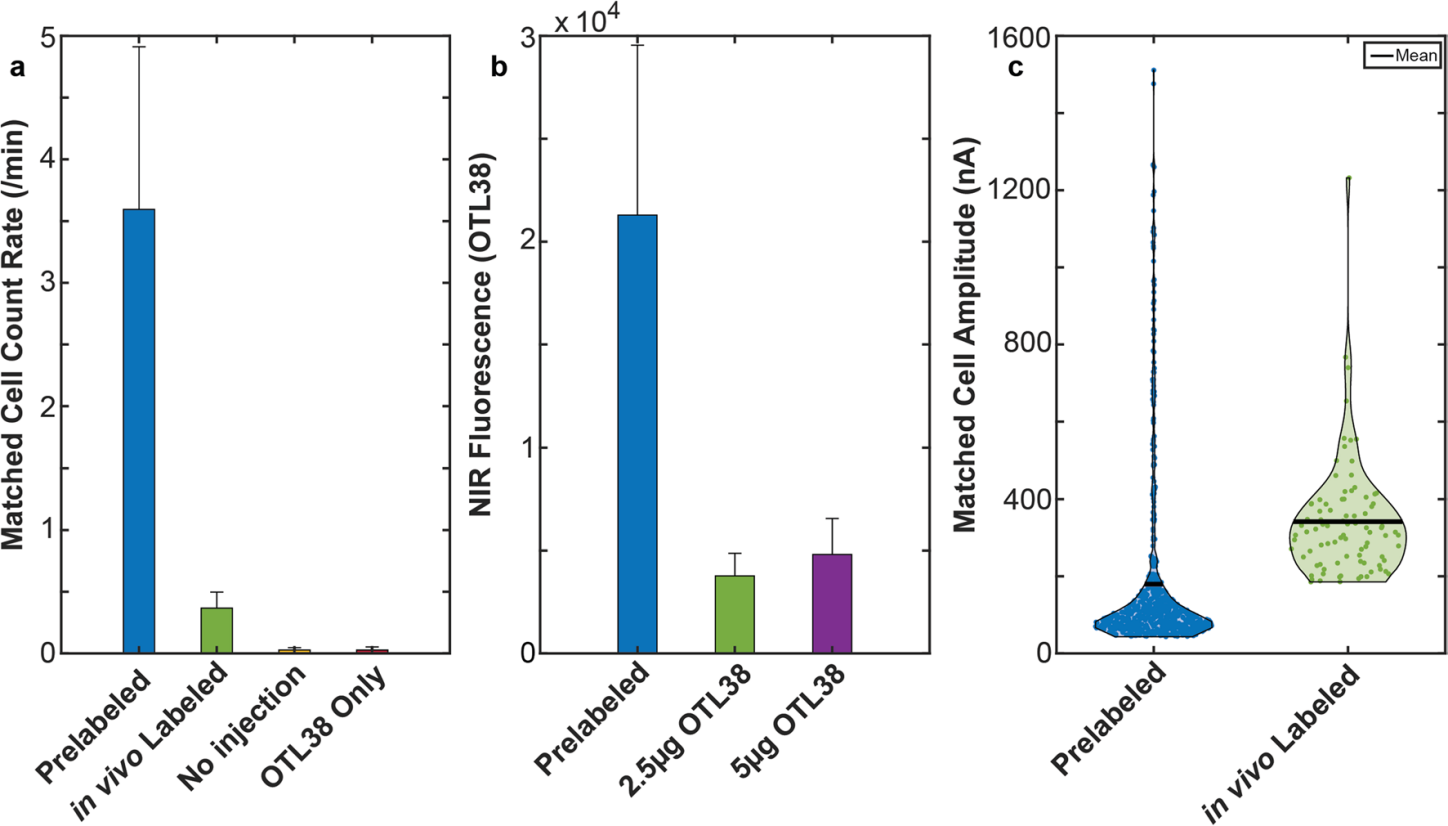
Comparison of prelabeled and *in vivo* labeled L1210A. **a** The NIR-DiFC count rate was significantly higher for mice injected with prelabeled L1210A cells than mice where L1210A cells were labeled in circulation (*N* = 3) **b** Fluorescence Flow Cytometry of L1210A cells found in mouse blood showed that prelabeled cells were brighter than L1210A cells labeled *in vivo* by injection of either 2.5 µg or 5 µg OTL38 (*N* = 3). **c** Analysis of measured NIR-DiFC in both cases showed that the peak amplitudes for prelabeled L1210A cells were higher than cells labeled *in vivo* by injection of 2.5 µg OTL38.

Although these experiments demonstrated that it is possible to label and detect CTCs *in vivo* with OTL38, as summarized in Fig. 5a, the measured count rate for *in vivo* labeled CTCs was 7.2 times lower than for prelabeled CTCs. Analysis of our data suggests that this lower count rate was due to two main factors which we consider in more detail here.

First, perhaps unsurprisingly prelabeled L1210A cells had higher mean uptake of OTL38 than L1210 cells labeled in circulation. The mean brightness of L1210A cells extracted from mouse blood labeled with each method is shown in Fig. 5b**.** Prelabeled L1210A cells were on average 5.6 times brighter than L1210A labeled *in vivo* with 2.5 µg OTL38 injected. Use of a higher injected OTL38 dose (5 µg) on another subset of *N* = 3 mice resulted on in only a modest increase in cell brightness, with prelabeled cells still on average 4.4 times brighter. Analysis of the measured NIR-DiFC peak amplitudes (Fig. 5c) for both cases showed that the maximum peak amplitudes were higher for prelabeled cells than for *in vivo* labeled cells. Somewhat unintuitively, the mean peak amplitude for the latter was 1.7 times higher than the former, which was due to the higher DiFC detection threshold (see below) and the fact that lower brightness cells were not detectable and therefore did not contribute to the mean.

**Fig. 6:**
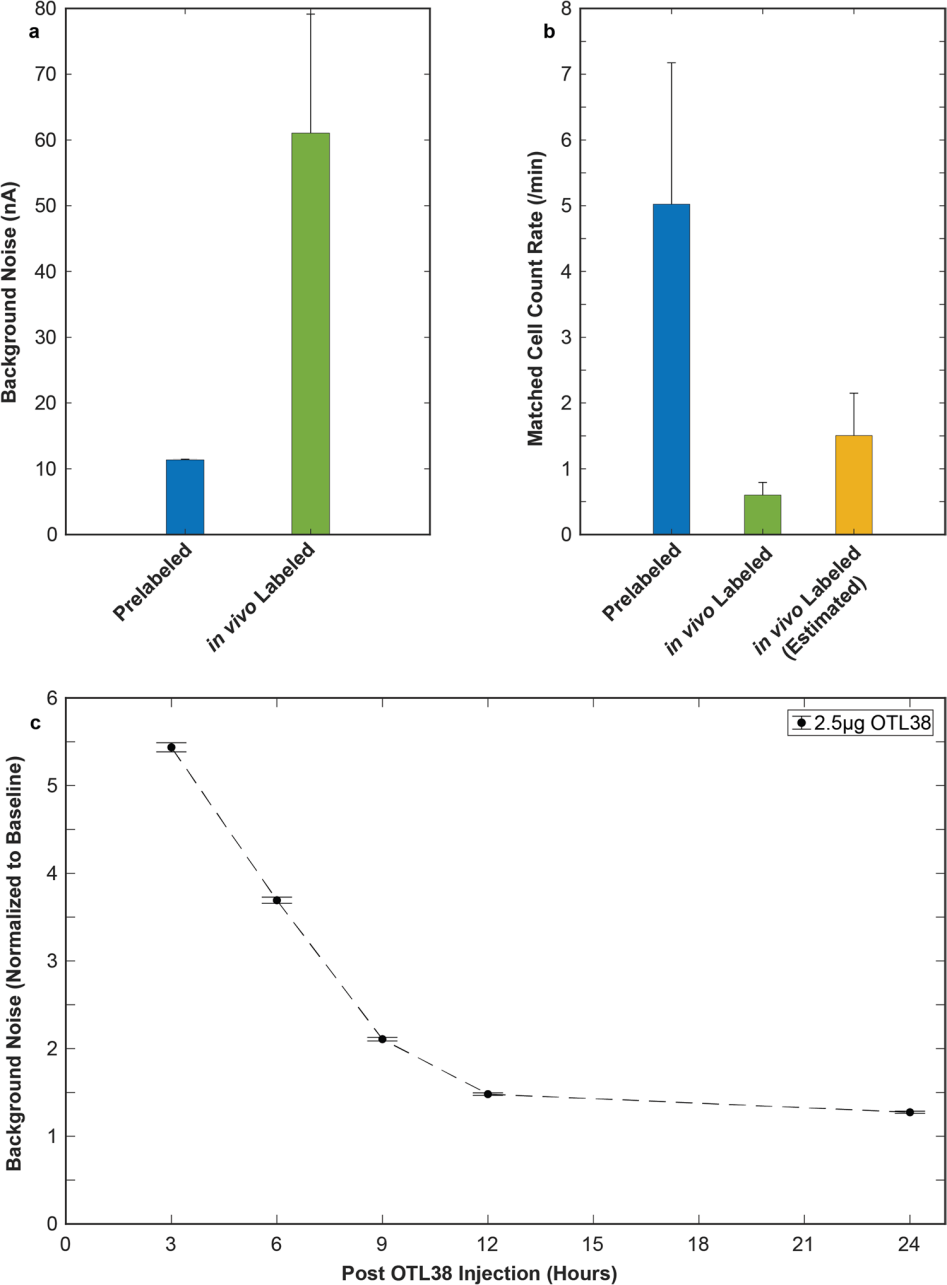
Fluorescence background and labeling efficiency of OTL38. **a** NIR-DiFC signal noise after background subtraction was on average 5.4 times higher for mice where 2.5 µg OTL38 was injected (“*in vivo* labeled”) compared to prelabeled L1210A cells (*N* = 3). **b** Based on flow data indicating that 30% of *in vivo* labeled cells would be detectable if the NIR-DiFC noise was the same as in the prelabeled case, the *in vivo* labeled count rate increased by a factor of 2.2. **c** The background signal (and associated background noise) decreased steadily after OTL38 injection returning to near baseline levels after 24 h.

Second, the NIR-DiFC background signal was significantly higher for mice injected with OTL38 compared to un-injected control mice or mice injected with prelabeled L1210A cells. Although we subtracted this mean in our data processing algorithm, the increased background resulted in an increase in noise which cannot be readily subtracted. Specifically, the NIR-DiFC background noise was on average 5.4 times higher for OTL38 injected mice than baseline (i.e. un-injected control mice or mice injected with prelabeled L1210 cells; Fig. 6a). We attribute this higher background noise to fluorescence originating from small amounts of OTL38 remaining in circulation or in the surrounding tissue. This noise both obscured fluorescence signals from less brightly-labeled cells and increased our detection threshold (which is 4 times the noise standard deviation) in signal processing, resulting in reduced detection of smaller cell peaks.

Analysis of NIR-DiFC data from both cases and fluorescence Flow Cytometry of cells measured in blood, we estimate that 30% of cells labeled by injection of OTL38 were of sufficient brightness to be detected with NIR-DiFC if this background noise was at baseline levels (Fig. 6b). Likewise, the estimated NIR-DiFC count rate would have been 2.5 times higher for cells labeled *in vivo* if the background noise was at baseline levels (Fig. 6c).

## Discussion

In this work, we demonstrated the feasibility of labeling circulating cancer cells *in vivo* with OTL38 and detect them with NIR-DIFC. Unsurprisingly, the number of cells detected (NIR-DiFC count rate) was lower for OTL38 labeled cells in circulation *in vivo* compared to cells prelabeled *in vitro* prior to injection. As above, our analysis indicates that this is partially due to less binding of OTL38 to CTCs in circulation compared to those labeled *in vivo* resulting in CTCs that are less bright.

Another major contributing factor was higher background signal due following injection of OTL38. The associated noise likely obscured less-labeled cells that would have been detectable (based on peak amplitude and fluorescence Flow Cytometry analysis) if background noise were at baseline (pre-injection levels).

Interestingly, in our previous work using the green fluorescence folate-receptor targeted probe EC17 we did not observe the same increase in background signal as we saw here [30]. We hypothesize that this may have been due to the higher attenuation of green light in tissue limiting the DiFC collection volume thereby reducing the autofluorescence from surrounding tissue [49, 50]. Moreover, OTL38 has been shown to clear rapidly from plasma with a half-life of < 30 min previously due to the small molecular structure [43]. As such, we speculate the much of the signal may have been from unbound OTL38 in surrounding tissue compartments.

Although this effect was problematic in the experiments here, our data also shows that this background signal subsides over time. Specifically, we continued to scan another subset of mice injected with 2.5 µg OTL38 for 24 h post injection and showed that the noise returned to near-baseline after 24 h (Fig. 6c). While this study was to show proof-of-concept for *in vivo* labeling and detection of CTCs, any clinical use of NIR-DiFC with OTL38 would likely be in conjunction with fluorescence guided surgery. In clinical FGS, the contrast agent is injected 3–24 h prior to surgery and binds and accumulates in the tumor [51–54]. As such, OTL38 labeled CTCs would be shed from the OTL38-labeled tumor, removing the need to label them while in circulation. Likewise, the increased background signal would subside due to the increased contrast agent and DiFC scanning interval.

Considering the current sensitivity levels of DiFC and OTL38, CTCs could in principle be detectable in human patients even at early stages of disease. As discussed above, a human DiFC system could be developed that would sample blood flowing through the wrist where blood flow rates are approximately 100 mL per minute. In human patients with metastatic disease, CTC numbers are typically observed in the range from 1 to 1000 CTCs per mL [11, 25, 29, 55]. Assuming a patient with 10 CTCs per mL of blood, with our current labeling and DiFC detection rate, a clinical DiFC system would in principle detect 10 CTCs/mL × 100 mL/min × 10 min × 30% sensitivity = 300 CTC counts in a 10 min scan. Although approximate, these suggest that our method could offer improved sensitivity over techniques using blood samples, and would allow measurement of changes in CTC numbers over time [25]. This, along with improved protocols for CTC labeling, is the subject of ongoing work in our lab.

## Conclusions

In summary, we showed for the first time that it is feasible to label circulating tumor cells directly in circulation *in vivo* using an FDA approved fluorescence molecular contrast agent OTL38. Use of our NIR-DiFC method allowed us to detect these CTCs externally from emitted fluorescence light in mice. Combined with further advances in DiFC instrument design and signal processing, this work may pave the way for potential human translation of DiFC in the future. In addition to further study of OTL38 with FR over-expressing tumors, fluorescence molecular contrast agents are being developed for clinical use which might have increased affinity for different tumor types. These clinical stage fluorescent molecular contrast agents can target cancer cells via surface receptors such as the epidermal growth factor receptor (EGFR), prostate specific membrane antigen (PSMA), and cathepsins which would provide the ability to target many different types of cancers [31, 56–58].

## Data Availability

The datasets generated and analyzed during this work and the MATLAB code are available through the Pennsieve data sharing platform (10.26275/btt6-puqw [https://discover.pennsieve.io/datasets/346]). Additionally, MATLAB Processing code can be found in a Niedre Lab GitHub repository (https://github.com/NiedreLab/OTL38-Labeling-and-Detection-of-L1210A-Cells).
